# Constructions of alcohol consumption by non-problematised middle-aged drinkers: a qualitative systematic review

**DOI:** 10.1186/s12889-018-5948-x

**Published:** 2018-09-18

**Authors:** Emma Muhlack, Drew Carter, Annette Braunack-Mayer, Nicholas Morfidis, Jaklin Eliott

**Affiliations:** 1 School of Public Health, University of Adelaide, Mail Drop DX 650 207, Adelaide, SA 5005 Australia; 2 School of Health and Society, University of Wollongong, Keiraville, NSW 2522 Australia

**Keywords:** Alcohol consumption, Qualitative, Systematic review

## Abstract

**Background:**

Current research into alcohol consumption focuses predominantly on problematic drinkers and populations considered likely to engage in risky behaviours. Middle-aged drinkers are an under-researched group, despite emerging evidence that their regular drinking patterns may carry some risk.

**Methods:**

We searched Scopus, Ovid Medline, and Ovid PsycInfo for peer-reviewed, English-language publications appearing prior to 31 December 2015 and relating to the construction of alcohol consumption by middle-aged non-problematised drinkers. Thirteen papers were included in our thematic analysis.

**Results:**

Middle-aged non-problematised drinkers constructed their drinking practices by creating a narrative of normative drinking via discourses of gender, identity, play, and learning to drink. They also used drinking norms to construct their gender and identity. Health was not identified as a significant consideration for the population of interest when constructing alcohol consumption, except where drinking behaviours were likely to harm another.

**Conclusions:**

These results suggest that public health campaigns aimed at reducing alcohol consumption may be more effective if they focus on unacceptable drinking behaviours instead of personal health outcomes.

**Electronic supplementary material:**

The online version of this article (10.1186/s12889-018-5948-x) contains supplementary material, which is available to authorized users.

## Background

Alcohol drinking and non-drinking is a complex social process influenced by a variety of factors and deeply embedded in the social environment. The current body of research into alcohol consumption focuses overwhelmingly on problematised drinkers such as young drinkers and binge drinkers. Low-level drinking is considered unproblematic in many alcohol studies (especially sociological studies) and in society more broadly [1]. In this sense, low-level drinking is non-problematised. However, low-level drinking can nonetheless be considered problematic since some alcohol studies (especially recent epidemiological studies) demonstrate that it presents health risks (increased all-cause mortality in the long term) [2, 3]. Thus, groups not previously considered problematic (which we henceforth refer to as non-problematised drinkers) can nonetheless be conceived of as drinking in ways that place them at risk.

One such group is middle-aged drinkers, whom we have defined as 30–65 year olds. While drinking in this age group has sometimes been problematised, such as in the case of alcoholism or binge drinking [4, 5], this group is rarely regarded as inherently problematic in the same way that youth drinkers are [6]. Nonetheless, these non-problematised drinkers may still drink in ways that impact negatively on their long-term health. For example, alcohol is a class 1 carcinogen with a dose response relationship and no known “safe” level of minimum drinking [7], meaning that regular drinking increases cancer risk. Daily drinking in Australia increases with age [8], and Australian middle-aged drinkers drink in excess of lifetime risk guidelines (no more than two standard drinks per day) [7] in similar proportions to young drinkers. Recent evidence indicates that 40–49 year-olds drink more alcohol than 18–24 year-olds [8]. In the United Kingdom, mean alcohol consumption (units/week) is highest for men aged 55–64 years and for women aged 45–54 years [9]. In the United States of America, a slight long-term (since 2002) downward trend in drinking frequency and quantity among adults under 25 years of age has coincided with a slight long-term increase in these measures among adults over 26 years of age [10]. The prevalence of high-frequency drinking tends to increase among older drinkers as they age, regardless of country, with the exceptions of Costa Rica, Nicaragua, and Uganda (where it is lowest among men aged 35–49) and Brazil, Ireland, and Kazakhstan (where it is highest among the same age cohort) [11].

Despite the increasing evidence that alcohol consumption among older drinkers is increasing over time, and that older drinkers are consuming more overall than younger drinkers, we know very little about the motivations and decision-making processes of non-problematised middle-aged drinkers when it comes to their alcohol consumption. We sought to fill this gap by systematically reviewing and synthesising qualitative literature that describes the ways in which non-problematised middle-aged drinkers construct their consumption of alcohol. Understanding alcohol consumption in this group will support more effective health interventions by, for instance, enabling health promotion campaigns aimed at reducing alcohol consumption among this group to speak to their greatest concerns and priorities.

## Methods

### Defining non-problematised alcohol consumption

The purpose of this study was to describe the constructions of the consumption of alcohol evident in academic analysis of accounts of non-problematised middle-aged drinking. We defined non-problematised alcohol consumption as alcohol consumption that is neither significantly harmful, nor socio-legally proscribed. We defined *significantly harmful* consumption as:

consumption that significantly increases the risk of ill health or injury to oneself or others, such as binge drinking, drink driving, drinking while pregnant, drinking after being diagnosed with a medical condition adversely affected by alcohol consumption (such as AIDS, Hepatitis, or CVD), and drinking that constitutes a substance use disorder (e.g. alcoholism and alcohol dependency).

We further defined *socio-legally proscribed* consumption as:

consumption that occurs against the constraints of prohibitive cultures (e.g. where religious conviction requires or strongly commends abstinence) or illegal consumption (e.g. where consumption is prohibited in specific locations).

We included studies that included alcohol consumption beyond recommended guidelines unless the drinkers either self-identified their drinking as problematic or it was presented by the authors as such.

### Study selection process

We searched three databases: Scopus, Ovid Medline, and Ovid PsycInfo, on the advice of the university’s discipline search specialist (Additional file 1). EM and NM also carried out hand-searches as described below. No additional papers were identified through hand-searching. The systematic review protocol was registered with Prospero (CRD42016032871).

We assessed studies for inclusion against the following questions:

1. Was this paper published in a peer-reviewed, English-language journal?
2. Did this study examine the consumption of alcohol as a beverage and examine how that consumption was experienced, understood, or discussed by participants, with regards to their own experience of alcohol consumption?
3. Did this study meet the required standards of data collection and analysis, e.g.: interviews or focus groups; use of participant voices; acceptable quality according to Critical Appraisal Skills Program (CASP) analysis?
4. Did this study include the population of non-problematised, middle-aged (30–65 year-olds) consumers of alcohol?

**Fig. 1:**
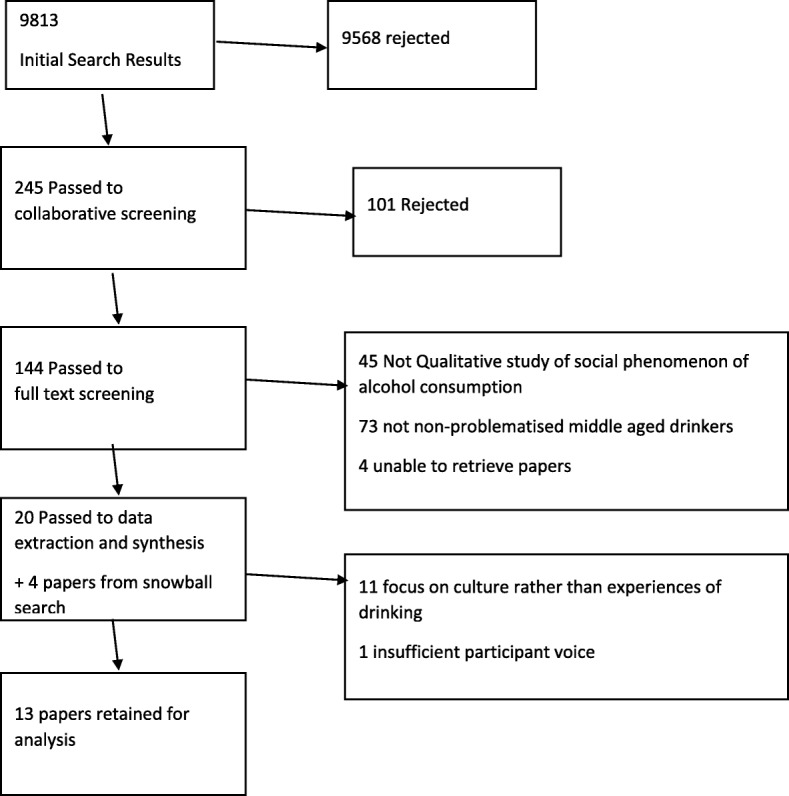
Study selection criteria

Papers that did not meet these criteria were excluded (see Fig. 1). Where papers met both exclusion and inclusion criteria (e.g. featured mixed-age participants or a combination of problematised and non-problematised drinking) only data pertaining to the aims of the study were included in the analysis.

An initial pool of 9813 search results was assessed by EM and NM against title, keyword and abstract. NM’s initial assessments (397 papers, determined by author surname A) were also reviewed by EM who identified no inappropriate exclusions, and so subsequent papers were checked independently by either EM or NM with a combined total of 245 papers for further analysis (Table 1).

**Table 1:** Examples of exclusion criteria

Criteria	Exclusion
1	Language other than English; published outside a peer reviewed journal
2	Biomedical effects of alcohol, alcohol in laboratory settings, animal tests; general perceptions of alcohol consumption; descriptions of “drinking cultures” and other high-level constructs that do not include individuals’ perceptions of their own drinking
3	Quantitative methods; opinion pieces; historical summaries; lack of participant voice.
4	Problematised populations or behaviours; participants solely <30yo or > 60yo

We checked exclusions and inclusions as separate stages due to the number of search results. The reviewers then met and together checked inclusions against the initial selection questions, to give a total of 144 papers.

EM and NM then independently assessed each paper on the basis of a full-text reading. Any disagreements were resolved through discussion, with 20 papers retained for data extraction and synthesis. EM subsequently hand-searched the publication lists of thirteen authors recommended by three field experts using a replication of the above process, with no additional papers identified. Finally, EM searched the reference lists of all papers included, with four additional papers identified to give a total of 24 included papers.

During the data extraction and synthesis phases, some papers were identified as inappropriate for the aims of the study [12]. Reasons for exclusion at this point were that the reviewed studies failed to meet inclusion criteria, as typified below:

- did not focus on the participants’ experience of drinking, but instead focussed on the cultural context in which drinking took place
- featured poor or unclear supporting data (e.g. assertions made without clear evidence to support them).

These papers were rejected as they did not meet inclusion criteria regarding participants’ participants own voices discussing their experience of alcohol consumption.

### Data Extraction & Synthesis

EM read and re-read each paper, extracting key findings using a data extraction form (Additional file 2). This customised form (modelled after recommendations found in Campbell et al. [12]) incorporated a modified version of the CASP Qualitative Checklist [13] as well as fields for extracting information about major themes and key findings of the papers. NM repeated this process on 6 papers as a check, with agreement on the CASP assessment and key findings of the papers. We placed key findings into a matrix with the papers’ pertinent metadata and coded them to themes, further identifying interactions between themes across the included papers. Themes were determined through EM and NM’s discussion of key results, identifying repeated elements between and within papers.

## Results

### Summary of papers

The majority of papers included in this review were from the UK (9), with Scotland (4) and England (4) being heavily represented. The remaining four papers are from Norway (2), Australia (1) and Japan (1). The Scottish papers had a specific focus on mid-life drinking. More information about the study aims, lenses of inquiry, populations, methods and key findings is appended in an attachment (Additional file 3).

### Principal findings

**Fig. 2:**
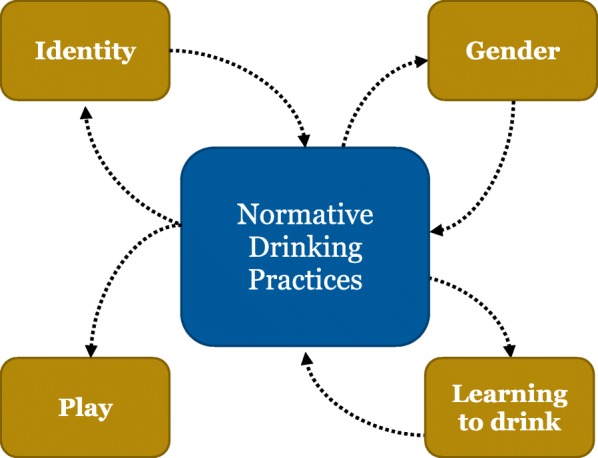
A model of themes which shape normative drinking

We found that middle-aged drinkers expressed understandings of normative drinking through the four interrelated themes of gender, play, identity, and learning to drink. These four themes shaped understandings of normative drinking in ways that also provided alternative interpretations of gender and identity (Fig. 2).

### Normative drinking

Normative drinking describes how people define both acceptable and unacceptable drinking practices. Acceptable drinking was framed as respectable drinking that was appropriate to one’s age or stage of life and which allowed participants to meet their responsibilities. By contrast, unacceptable drinking was drinking that was inappropriate to one’s age or stage of life and/or prevented one from meeting their responsibilities.

Acceptable and unacceptable drinking practices were defined by numerous factors, including the presence or absence of certain behaviours in public and effects on the drinker. Across the dataset, we consistently found that participants stated that drinkers should neither experience nor display any negative effects of their drinking, such as slurred speech, vomiting, an unsteady gait, or a hangover the next day [14]. In Ling et al. [15], participants associated these effects with people who had problems with drinking, or with young drinkers: one participant described “these young teenagers on the streets can’t walk, sort of like collapsed in a heap cos they’ve drank that much” [15]. Being able to meet work and domestic responsibilities was also frequently mentioned, especially in studies among parents and caregivers [16, 17]. Another important factor in determining acceptable and unacceptable drinking was that others should not suffer as a consequence of a person’s drinking: thus, drink-driving was always constructed as unacceptable [15], and responsible parenting required that parents limit alcohol consumption [16].

The boundaries between acceptable and unacceptable drinking were also described in terms of being appropriate or inappropriate to one’s age and stage of life. For some participants, cosmetic issues such as weight, appearance, and premature ageing [17, 18] were deemed important in determining appropriate levels of consumption. For example, participants in Lyons et al. [17] described how their ageing bodies responded to alcohol, such that avoiding the negative effects of drinking required them to consume less and to closely monitor their own bodily response to alcohol. Similarly, some mothers in Killingsworth’s ethnography said that growing older meant that respectable behaviour involved drinking less; they were pleased to discover that a mutual acquaintance was pregnant, since this would force her to drink less and thus better conform to their notion of acceptable and respectable middle-aged parenting behaviour [19].

Ling et al. [15] described a way of determining acceptable drinking whereby participants defined a “safe” level of alcohol consumption according to their own experiences, actively rejecting as irrelevant government drinking guidelines and public health messages (apart from messages around drinking and driving). For example, one participant stated, “I’ve seen all the education, I don’t think I drink excessively but if you put me on a scale according to the Government I am off the scale but, I feel fit, healthy …” [15]. This positioned his experience of *feeling* fit and healthy as the authoritative determinant of acceptable drinking, not Government statements. Some participants explicitly described particular drinking behaviours as healthy; they described red wine as being good for the heart and circulation [14] and men drinking together as good for mental health [1]. By contrast, Brierley-Jones et al. [18] reported that drinkers in the traditional pub settings were indifferent about any link between alcohol and health and were likely to “see the relationship between alcohol and future health, in nihilistic terms, as something largely outside of their control,” describing it as being “like a lottery” [18].

Acceptable drinking further differed depending on location. Brierley-Jones et al. [18], utilising Bourdieu’s concept of habitus,^1^ described how two locations gave rise to differing acceptable practices, distinguishing between the patterns of the “home” and “traditional” habitus of drinking. The former was associated with moderate consumption of wine throughout the week, while the latter was associated with more expansive consumption of beer and/or spirits in pubs on weekends.

Normative drinking was also context-driven: the same behaviour could be acceptable in one context, but unacceptable in another. For example, in Nesvåg and Duckert [20], work-related drinking featured a transition from formal to informal or social phases of an event, with differences in acceptable drinking practices. Applying informal drinking practices to the formal phase was considered by participants to be a *faux pas*, which the authors described as carrying a “risk of being marginalised” [20].

### Gender

Part of what made drinking acceptable or unacceptable in these studies was how drinking patterns adhered to gendered expectations of behaviour. Drinking practices were used as a tool to express and display adherence to and transgression of gender norms. What and where participants drank also mattered. For example, in several studies from the United Kingdom, certain drinks were considered appropriate for women and others for men [1, 16, 17, 21], and domestic drinking was associated with women, public drinking, with men [1].

In Holloway et al. [22], some female participants challenged particular gendered drinking norms but reaffirmed others. For example, respondent Audrey, comfortable going to pubs herself, related that “… I find it a bit odd in this day and age, I know there are still women that I know who wouldn’t meet you in a pub, and certainly wouldn’t go to the bar, and some people locally their husbands always buy the drinks” [22]. Nonetheless, although challenging the norm that pubs are for men, she was more permissive of male drinking, saying that she felt “less negatively towards a drunk man than I do towards a drunk woman” [22].

As mentioned earlier, men also experienced constraints on their drinking: whilst men were less scrutinised in how much they drank, they were nonetheless constrained in what and where they could drink. This is not to say that men could not move outside gendered norms of drinking: in Emslie et al. [1], participants Graham, Ewan, and Hugh drew on the social capital of wine connoisseurship to construct alternate masculinities, and other men stated that drinking outside of the ‘pints in pubs’ model could be done in “exceptional circumstances” such as holidays and special occasions. [1].

Emslie et al. [1] also showed how adherence to some gendered norms of drinking allowed the transgression of other gendered norms. They described how men’s adherence to a very masculine model of alcohol consumption of pints at the pub enabled men to do un-masculine “emotional labour” (i.e. talking about feelings) around mental wellbeing [1]. Here, the un-masculine work of talking about feelings was counterbalanced by the highly masculinised model of drinking.

### Identity

In these studies, identity was important for constructions of acceptable and unacceptable drinking. How people drink both contributes to their identity, and is shaped by the identity they have crafted for themselves. For example, Ho [23] found that the display of alcohol-related knowledge (whilst drinking) for white-collar women in Japan was “useful for enhancing their image as corporate executives in business dealings, in addition to projecting themselves as cosmopolitan individuals” [23]. Thurnell-Read [24] further reported that participants in his study “thought of and spoke of themselves as ‘ale drinkers’”, with routines peculiar to the identity of ale drinkers enacted only when ordering and drinking an ale with other members of the Campaign for Real Ale (CAMRA)^2^ [24].

Alcohol consumption was also regarded as a way of reclaiming past identities, or transitioning from one identity to another. In Ling et al. [15], drinking was described by one male participant as a way of reclaiming his identity before parenting by “[making] you feel like an adult again” [15]. Women in the same study described drinking as a way of “reliving [their] youth”, observing how particular drinks were associated with earlier identities [15].

### Play

Normative drinking was closely tied to ideas of lay: in many papers, alcohol consumption signalled the cessation of work or responsibilities, and also a social or leisure activity in its own right.

We noted the use of alcohol as a marker of the boundaries of work or responsibility across cultures. Commencing drinking was described as a way of declaring that work or other responsibilities were completed and that recreation and relaxation had begun [16, 17, 21]. Alcohol was also described as being instrumental in creating the state of post-work relaxation [22].

Drinking norms specific to social settings can also be seen in the relationship between guest and host. Holloway et al. [22] described how “complex systems of sociality, hospitality and reciprocity” led participants, even non-drinkers, to feel compelled to keep a variety of alcoholic beverages about the house in order to fulfil the role of host [14]. Emslie et al. [16] described the difficulties of maintaining appropriate drinking behaviour in home settings, where acceptable drinking practices can be paradoxical: participants in their study described how a good host ensures that guests’ glasses are constantly topped up, constituting pressure to drink, but (as we highlighted earlier) drinking norms dictate that drinkers avoid obvious signs of drunkenness. Thus, we note that the duty of the guest to consume the constantly refreshing supply, flowing from the largesse of the host, is incompatible with the guest’s duty to exercise self-control. Participants in the 2012 study by Emslie et al. [16] negotiated this paradox through the provision of suitable excuses to limit or avoid drinking, such as detoxing or being on a diet.

Alcohol consumption was described as a focussed leisure activity by Thurnell-Read [24]. His study of “Real Ale Enthusiasts” showed that, rather than being a signal of relaxation, the consumption of alcohol and the connoisseurship around it functioned as a recreational activity in its own right [24]. Participants in the study explicitly contrasted their CAMRA drinking with “normal” drinking, and one described having to “watch yourself sometimes” to ensure that the serious leisure activity of CAMRA connoisseurship did not spill over into social time spent with those who are “into their beers but not like I am” to avoid “feel [ing] like a daft prat” [24].

### Learning to drink

The final theme apparent in these studies is the idea that normative drinking is something that people learn, both from family and culture as well as from knowledge of personal preferences and the effects of alcohol on them. This involved learning the “skill” of non-problematised drinking, as well as learning about alcohol and how to display that knowledge as part of normative drinking behaviours.

Illustrating how drinking patterns could be learned from family heritage and local tradition, Brierley-Jones et al. [18] demonstrated how the reproduction of traditional drinking habitus established a connection between present-day white-collar drinkers and a blue-collar history of family and community. By drinking in the village pub that used to service the foundry, drinkers in the traditional habitus could align themselves with this blue-collar history “despite the non-physicality of white-collar work” [18]. Drinkers in both the home and traditional habitus described the importance of their parents’ behaviours in establishing their own drinking behaviours and attitudes. The authors detailed how a taste for particular beverages could be acquired, with one respondent explaining how one would begin to drink as a social act and then “you start liking it” [18]. Finally, Lyons et al. outlined how participants’ personal limits on alcohol were learned from past experience, with the bodily experience of alcohol consumption becoming “so well-rehearsed that they no longer require conscious intervention or scrutiny” [17].

### Interactions between themes

Gender, identity, play, and learning to drink each help to define normative drinking. In addition, as we explain below, they interact with each another to build a more complex picture of the nature of normative drinking.

It is clear from these studies that gendered norms of drinking affected the mode of play. For example, for men within Lyon’s et al. study, “drinking alcohol provided embodied pleasure as a reward for working hard” [17]; for women, drinking with friends was an acceptable way to relax and take time away from domestic responsibilities (e.g. housework, childcare) and, for some women, away from paid employment [17]. In Emslie’s study, however, the separation from responsibility was incomplete as women still “retained the main responsibility for their children” [21]. For these women, acceptable drinking practices were constrained by “the effect on children if they saw their mothers drinking (excessively),” [21]. These gendered expectations worked to limit the extent to which women were able to relax.

In other studies, the interaction between gender and normative drinking served not simply to limit, but also to prevent opportunities for play. Holloway et al. [22] described how participant Doris (a widow) was excluded from some social opportunities due to her gender and age, given perceptions that “it’s not seemly for a woman of [her] age to walk down and go in to the pub on her own” [22].

The interaction between gender, play, identity, and normative drinking was also evident in the work environment. Female managers in the study of workplace culture by Nesvåg and Duckert [20] felt constrained by gendered and work-specific drinking norms, with one woman stating that “in company organised parties I feel my way of drinking is a part of the management performance,” a clear contrast to the nature of alcohol consumption as play [20]. In a study of female managers in Norway by Buvik and Sagvaag [25], the interaction of these themes served to limit alcohol consumption. Alcohol was so strongly associated with relaxation that the women interviewed were reluctant to drink in the work environment because it could undermine the control that they were expected to maintain, both as women and as managers. Their visible status as managers and women created a restrictive environment that limited acceptable drinking practices. While women could take the opportunities for recreation and relaxation afforded by alcohol consumption, this was either (1) in a home environment, (2) with other managers, or (3) with more restrictive limits on acceptable behaviour than those encountered by male colleagues. Some participants stated that they would rather forgo workplace drinking entirely in order to fulfil gendered care-related duties [25]. As noted above, Emslie et al. [21] similarly reported that some women experienced incomplete separation from their domestic duties, which prevented them from fully engaging in recreation and relaxation activities. Ultimately, drinking practices that were otherwise acceptable in the work context were restricted by gendered expectations of the roles of manager and mother. However, the authors described attempts by some women to move beyond these restrictions with nights out, when they escaped “from their work and domestic responsibilities” [21] and followed new drinking norms that allowed them to resolve “multiple co-existing femininities while keeping a coherent sense of one’s self and identity” [21].

Just as gendered norms of drinking affected the mode of play, so gendered drinking norms could construct an identity that moved beyond simplistic binaries of gender-appropriate behaviour. A study of playgroup mothers in Australia showed how they consumed and discussed alcohol in ways that skirted the edges of gender expectations without actually transgressing them: the mothers drank, but not too much, or they talked about drinking, rather than actually drinking [19]. In this way, the women simultaneously reinforced, and resisted, “dominant, relatively traditional notions of (female) gender and motherhood” [19]. Ho [23] later described how, in Japan, women in white-collar professional employment participated in the recreational practices of drinking and host clubs (an environment historically restricted to men, and still somewhat gendered) to define themselves as *sarariman* (white-collar professional workers). One study participant consumed masculinised drinks such as beer and whisky to redefine her identity: by transgressing gendered drinking norms, she adopted masculinised traits that enhanced her identity as a (female) manager in charge of a male-dominated sales team [23]. Similarly, Emslie et al. [21] described how their participant ‘Madeline’ used masculinised drinking practices (“playing the lad”) [21] when drinking with male colleagues in defiance of gendered drinking norms. The authors hypothesised that Madeline used the counter-balancing resources of her class position to “construct herself as (respectably) feminine” and legitimise her ‘masculine’ drinking.

Participants in various studies used the learned aspects of normative drinking to construct their identity. In Emslie et al. [1], connoisseurship, namely the display of learned knowledge and appreciation of (in this case) alcoholic beverages, was used as a form of social capital in establishing a cultured identity. By deploying knowledge of wine and malt whisky, participants in two all-male focus groups could “position themselves as ‘accomplished individuals’ in the social hierarchy through this demonstration of taste and discernment” [1]. By contrast, in Holloway et al. [14] many respondents negotiated identities through a considered *rejection* of connoisseurship. By declaring a preference for mid-range wines and simultaneously repudiating the label of aficionado, respondents successfully navigated around the possibility of being seen as pretentious while still accessing the cultural capital associated with wine consumption [14].

Group identities were also constructed through how individuals learnt to drink. In Nesvåg and Duckert [20], the “knowledge and communication” of various characteristics of alcoholic drinks were strongly tied to a continental European cultural ideal valued by the management of an oil company. This company norm and identity then influenced the drinking behaviours of individuals, with contrary behaviours (e.g. obviously succumbing to drunkenness) being minimised and/or denied by individual workers [20].

### Limitations

Most papers we reviewed reported studies carried out in the Anglosphere (predominantly the United Kingdom), possibly because of our inclusion requirement that publications be in English. Thus, our findings may not have captured all research around middle-aged drinking and may be limited in cross-cultural applicability.

Several reviewed papers shared authorship or a data source. We regarded papers from the *Drinking Attitudes in Midlife* (DrAM) study [1, 16, 17, 21] as closely linked to one another due to the common data pool, authorship, methods, and theoretical perspective. Two papers examining the geographies of alcohol featuring common authorship, data, topic, and lens of inquiry [14, 22] were moderately linked to one another. Another two papers [15, 18] we regarded as slightly linked to one another due to significant overlap in authorship.

## Discussion

Alcohol drinking and non-drinking is a complex social process that is influenced by a variety of factors and deeply embedded in the social environment. Based on the literature, we have articulated five themes that explain how non-problematised middle-aged drinkers construct their drinking. Participants in reviewed studies distinguished between acceptable and unacceptable drinking practices in nuanced ways to produce their version of normative drinking. Normative drinking was influenced by play, gender, identity, and learning to drink, as well as by interactions between these concepts.

Our results offer insights into how public health messages about the health effects of alcohol consumption may be received by middle-aged non-problematised drinkers, and the barriers that may prevent this group from receiving and acting on these messages. In Ling et al. [15], public health messages were subordinated to subjective experience in individuals’ determinations of healthy drinking behaviours. In Holloway et al. [14] and Emslie et al. [1], competing discourses of alcohol and health allowed alternative definitions of healthy behaviour. In Brierley-Jones et al. [18], health messages were rejected entirely, replaced instead by a fatalistic view of health. Collectively, these findings suggest that, for middle-aged drinkers, what makes drinking safe and acceptable is determined by whether the drinker can still meet responsibilities and adhere to socially expected models of behaviour. This suggests that the principal barrier to reductions in alcohol consumption is not the lack of information about health risks. The drinkers in these studies were aware of public health messages, but drew upon alternative narratives to reframe their behaviours in ways that minimised or dismissed personal risk. Health was either described as a minor concern or not considered at all.

We have shown how participants maintain their status as non-problematised drinkers partly by setting boundaries around drinking behaviours and adhering to certain norms. There are some parallels between these practices and the normalisation of substance abuse by drug users. For example, “non-problem” drug use is normalised [26–28] and “otherwise law-abiding citizens have collectively socially reconstructed an illegal act” when considering the distribution of drugs among networks. [29] Drinkers and drug users employ similar strategies to ensure that they remain on the “right” side of the line demarcating problem behaviours: they ensure that the physical environment minimises physical risk [30] and they distance their own (potentially problematic) actions from those of the problematic (be it binge drinker or drug dealer) [28]. It is the identity which determines problematisation, rather than the behaviour. If one is not a binge drinker or a drug dealer, then one’s drinking or one’s drug use is not problematic. The similarities between these two groups may provide a broader understanding of the ways in which non-problematised drinkers approach their drinking and associated behaviours.

Several themes in this paper may be helpful when formulating interventions to limit or moderate alcohol consumption. Narratives of unacceptable drinking practices may be useful in framing public health messaging of relevance to this demographic. For example, public health strategies can focus on meeting responsibilities to others, the possibility of causing harm to others, the requirement for respectability in drinking, the physical limits of ageing bodies and subsequent physical consequences, and gendered expectations of behaviour. One example of a campaign using notions of respectability and behaviour appropriate to one’s age and stage of life is the Motor Accident Commission of South Australia’s “Drink Driving—Grow Up” campaign, which relies on notions of moderate drinking being respectable, and excessive drinking being inappropriate for mature adults, when it suggests that drink-driving is ‘childish’ behaviour by using child actors in adult roles [31].

How people define themselves as drinkers—their identities—can also be used in public health campaigns. Some identities, such as the connoisseur and the “real ale drinker,” are closely tied to consumption—without consumption, the identity does not exist. However, these specific identities are concerned with a specific type of drinking, rather than with consuming a lot of alcohol. These identities could be framed in ways that reject higher levels of consumption and emphasise quality over quantity in consumption. There is a danger, however, that this message of gaining social capital through moderate drinking could be exploited. For example, the DrinkWise “Drinking: Do It Properly” campaign was promoted as influencing “young adults (18-24) to drink responsibly – by moderating the intensity and frequency of binge drinking occasions” [32]. However, the campaign received strong criticism as promoting drinking, rather than moderation in drinking [33, 34].

It is also possible for the themes we have identified in this paper to be used in public health campaigns in ethically problematic ways. For example, the use of gendered public health messaging to encourage or discourage particular modes of consumption is problematic, since many of the gendered drinking norms are closely tied to traditional and potentially oppressive notions of masculinity and femininity. For example, the “Think Twice” campaign of Balance (the North East UK Alcohol Office) and Breakthrough Breast Cancer included an image of two glasses of rosé swirled to resemble a woman’s cleavage. [35] This image relies on a gendered form of alcohol consumption, which may, in turn, reinforce wider gender stereotypes.

In contrast to this, the DrinkWise “Kids Absorb your Drinking” campaign [36] used parental identity and the learned nature of alcohol consumption. We demonstrated that drinking norms for parents are gendered, with the assumption that women undertake the bulk of childcare and domestic responsibilities. This campaign avoided gendered expectations of parental responsibility by invoking the father-son relationship. In this way, the campaign did not exploit traditionally gendered parenting roles while still using themes of normative drinking in a way that challenged drinking behaviours. It is important to note, however, that relying upon a traditional educational model of intervention is insufficient; as noted, messages tended to be subordinated according to drinkers’ own experiences. In this regard, it may be more useful to use these findings to shape and influence public debate to bring about legislative and regulatory changes that create a safer drinking culture. This has been done to great effect with tobacco, another non-problematised substance that was denormalized and problematised as part of an ongoing campaign to reduce lung cancer. However, caution is advised in denormalising and problematising. Ethical issues around the potential for stigmatisation, seen in tobacco smokers, must be considered when implementing campaigns such as this so that we do not unacceptably cause harm in our search to do good.

## Conclusion

For middle-aged non-problematised drinkers, alcohol drinking and non-drinking is a complex social process that is influenced by a variety of factors and deeply embedded in the social environment. We found that middle-aged drinkers constructed their alcohol consumption within a framework of Normative Drinking. This key concept was expressed through the four interrelated themes of Gender, Play, Identity, and Learning to Drink. Normative drinking was also used to offer alternative interpretations of gender and identity.

Concerns about health and healthy behaviour, however, were either minor or non-existent. For these drinkers, alcohol was both a tool for relaxation described by learned norms of behaviour around gender and identity, and a means by which the self could be expressed through deliberate adherence to and rejection of those norms. This offers possible narrative frameworks for public health interventions around alcohol consumption, although care must be taken to ensure that ethically problematic issues around gender and identity are considered.

## Additional files

Additional file 1: Search matrix exemplar. (DOCX 16 kb)

Additional file 2: Evaluation & extraction tool. (DOCX 15 kb)

Additional file 3: Metadata and paper summaries. (XLSX 16 kb)

## Abbreviations

CAMRA: Campaign for real ale
CASP: Critical appraisal skills program

## Footnotes

1 “…the sum of social structuring influences on individual biographies that direct aesthetic choices below the level of consciousness”, “…a set of dispositions” that “secures both the patterned and the durable nature of practices such as eating and drinking.” [18]

2 The Campaign for Real Ale (CAMRA) is a society based around a connoisseurship approach to consuming “real ale”, or cask-conditioned beer, with an associated stereotype of members as “bearded, unfashionable, elderly, and boorish” [24]
