# Supplementary material for: Constructions of alcohol consumption by non-problematised middle-aged drinkers: a qualitative systematic review
Source: BMC Public Health. 2018 Sep 18;18:1016. doi: 10.1186/s12889-018-5948-x (PMC6142397; doi:10.1186/s12889-018-5948-x)
Supplement: Supplementary file 1 — Search matrix exemplar. (DOCX 16 kb) [file 12889_2018_5948_MOESM1_ESM.docx]

| **Within 3 words** | | **AND NOT** | |
| --- | --- | --- | --- |
| **ALCOHOL** | **CONSTRUCTION** | **EXCLUSIONS (title, key)** | |
| alcohol  OR drinking  OR drinker*  OR beer  OR wine  OR liquor | “pattern*”  OR “consumption”  OR “practice*”  OR “attitude*”  OR “identi*”  OR “social”  OR “sociali?ation”  OR “socially”  OR “norm*”  OR “cultur*”  OR “perception*”  OR “experience*”  OR “context*”  OR “motivat*”  OR “account*”  OR “moderat*” | Alcoholism  OR chronic  OR heavy drink*  OR drunken*  OR abus*  OR problem*  OR dependenc*  OR tobacco  OR smok*  OR nicotine  OR marijuana  OR pregnan*  OR f*etal  OR f*etus  OR diabet*  OR cirrho*  OR HIV  OR AIDS  OR cancer*  OR cardiovascular  OR disease* | OR injury  OR assault*  OR violen*  OR crim*  OR aggress*  OR depress*  OR “mental illness”  OR drug*  OR risk  OR risky  OR hazard*  OR adolescen*  OR “controlled study” |
