# Supplementary material for: Constructions of alcohol consumption by non-problematised middle-aged drinkers: a qualitative systematic review
Source: BMC Public Health. 2018 Sep 18;18:1016. doi: 10.1186/s12889-018-5948-x (PMC6142397; doi:10.1186/s12889-018-5948-x)
Supplement: Supplementary file 2 — Evaluation & extraction tool. (DOCX 15 kb) [file 12889_2018_5948_MOESM2_ESM.docx]

Qualitative Systematic Review: Quality Assessment and Data Extraction Tool

## Bibliographic Details

| *Title* |  |
| --- | --- |
| *Author(s)* |  |
| *Journal Title* |  |
| *Vol/Issue/pg/yr* |  |

# Quality Assessment

## Is there a clear statement of research goals?

What is the stated research goal? How is this research important?

## Is the methodology appropriate?

Has the methodology been outlined? Is it appropriate to the research goal?

## Is the research design appropriate to the aims?

Has the research design been justified/is there discussion of the method selection process?

## Is the recruitment strategy appropriate to the aims?

How were participants selected? Why were these participants appropriate to the aims? What additional background information is given about recruitment? What data is given about participants? (NB check alcohol consumption levels)

## Was data collected in a way that addressed the research issue?

Is it clear how data was collected? Have collection methods been justified? Have methods been made explicit? Were methods modified, and if so then why? What form was data collected in? Is saturation discussed?

## Has the relationship between researcher and participants been adequately considered?

Has the researcher critically considered their own role and potential bias/influence at any stage of data collection and analysis?

## Have ethical issues been considered?

Was ethical clearance given for the project? Are any of the “mechanics” of ethics (e.g. consent, data storage, confidentiality) discussed?

## Was data analysis sufficiently rigorous?

Is there an in-depth description of the analysis process? Is it clear how any data presented was selected (i.e. quotes)? Is there sufficient data presented to support findings? Are contradictory data taken into account? Is reflexivity factored into analysis?

## Is there a clear statement of findings?

Are findings made explicit? Is credibility addressed? Are findings discussed in relation to the research question? Is evidence for/against researcher arguments discussed (if appropriate)

## How valuable is the research?

What is the contribution to current knowledge or understanding? Are new areas of research identified? Are results transferable or useable in other ways than the initial intention?

# Data Extraction

## Major Findings & Conclusions

|  |
| --- |
|  |
|  |
|  |
|  |
|  |
|  |
|  |
|  |
|  |
|  |
|  |
|  |
|  |
|  |
|  |
|  |
|  |
|  |
|  |
|  |
|  |
|  |
|  |
|  |
|  |
|  |
|  |
|  |
|  |
|  |
|  |
|  |
|  |
|  |
|  |
|  |
|  |
|  |
|  |
|  |
|  |
|  |
|  |
|  |
|  |
|  |
|  |
|  |
|  |
|  |
|  |
|  |
|  |
|  |
|  |
|  |
|  |
|  |
|  |
|  |
|  |
|  |
|  |
|  |
|  |
|  |
|  |
|  |
|  |
|  |
|  |
|  |
|  |
|  |
|  |
|  |
|  |
|  |
|  |
|  |
|  |
|  |
|  |
|  |
|  |
|  |
|  |
|  |
|  |
|  |
|  |
|  |
|  |
|  |
|  |
|  |
|  |
|  |
|  |
|  |
|  |
|  |
